# Enaminonitriles in Heterocyclic Synthesis: A Route to 1,3-Diaryl-4-aminopyrazole Derivatives

**DOI:** 10.3390/molecules18010535

**Published:** 2013-01-02

**Authors:** Hanadi Y. Medrasi, Mariam Abdullah Al-Sheikh, Abdellatif Mohamed Salaheldin

**Affiliations:** 1 Department of Chemistry, Sciences faculty for Girls, King AbdulAziz University, Jeddah, P.O. Box 13343, Jeddah 21493, Saudi Arabia; 2 Department of Chemistry, College of Applied Sciences, Umm Al-Qura University, P.O. Box 13401, Makkah 21955, Saudi Arabia

**Keywords:** enaminonitriles, 4-aminopyrazole, Thorpe-Ziegler cyclization, microwave irradiation

## Abstract

Benzylcyanide and 4-nitrobenzylcyanide condensed with triethyl orthoformate and piperidine or morpholine to yield 2-aryl-2-piperidinyl or 2-morpholinylacrylonitriles. These coupled with aromatic diazonium salts to yield the 2-arylhydrazno-2-arylethane nitriles in good yields. The latter were converted into 4-aminopyrazoles in good yields using the Thorpe-Ziegler cyclization.

## 1. Introduction

**Figure 1:**
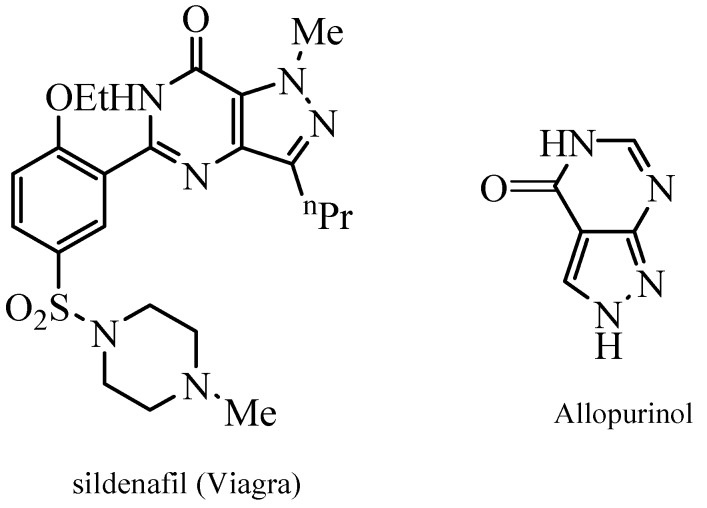
Structure of Viagra and Allopurinol.

**Scheme 1:**
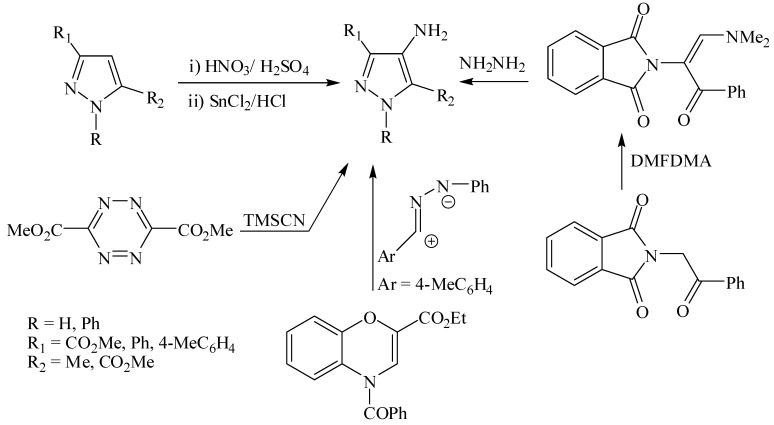
Literatures preparation of 4-aminopyrazole derivatives.

The synthesis and chemistry of 4-aminopyrazole-5-carboxylic acid derivatives is now receiving considerable interest [1,2,3,4]. Potential applications in pharmaceutical industry are most likely behind this interest. For example, ethyl 3-butyl-4-aminopyrazole-5-carboxylate is a key intermediate in synthesis of Viagra (Figure 1) [5,6] while 5-aminopyrazole-4-carboxamide is the building block for the synthesis of allopurinol (Figure 1). 4-Aminopyrazoles has been generally prepared via nitration of pyrazoles and subsequent reduction of the formed 4-nitropyrazole but such approach requires discarding large amounts of hazardous acid waste [1,2], which could be minimized as recently reported [7]. The ring transformation reaction of 1,2,4,5-tetrazines to 4-aminopyrazoles by cyanotrimethylsilane (TMSCN) [8]; addition of nitrile imines to benzo-1,4-oxazines [9] or reaction of ethyl diazoacetate with benzyl cyanide are alternate routes to 4-aminopyrazoles [10], but again these either utilize explosive starting materiales or rather expensive ones. A route to 4-aminopyrazoles via a Gabriel-like synthesis has also been recently reported, but this approach is not atom economic and also employs expensive dimethylformamide dimethylacetal (cf. Scheme 1) [11].

Microwave-assisted reactions have received great interest in synthetic organic chemistry. Microwave heating has been employed as a frequent resource for improvement of classical reactions. The major benefits of performing reactions under microwave condition are: shorter reaction times, ease of isolation of the products after easy work-up, significant rate enhancements and higher product yields as compared to reactions run under conventional heating [12,13,14].

## 2. Results and Discussion

**Scheme 2:**
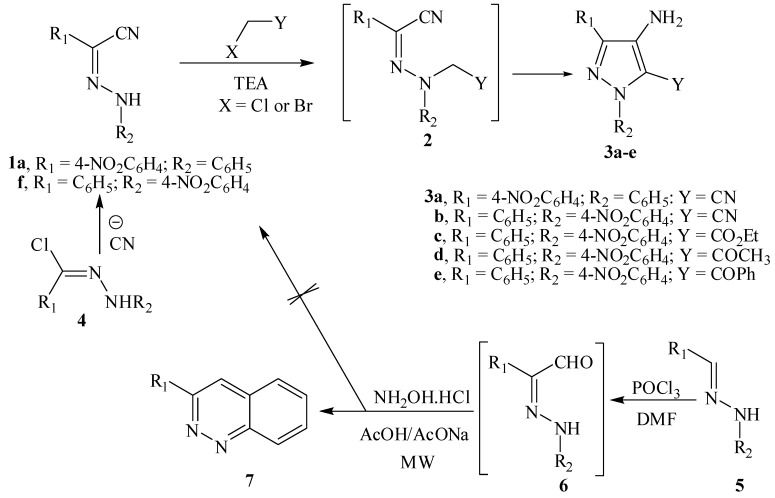
Preparation of 4-aminopyrazoles.

In conjunction to our interest in chemistry of pyrazoles [15,16,17] and condensed pyrazoles [18,19,20] we decided to develop an efficient route to the title compounds. A logical route to these compounds would be the reaction of hydrazones **1a**,**f** with functionally substituted alkyl halides to yield targeted compounds **3** *via* intermediate **2**. Although **1a**–**c** can be readily obtained from reaction of **4** with cyanide ion following a procedure similar to that described earlier [21], this approach was discarded based on the apparent hazards. We firstly considered the reaction of aldehyde hydrazones **5** as a route to **6** that can then be converted to **1** via reaction with hydroxylamine in a microwave oven as has been recently reported from our laboratories. However, under such conditions cinnolines **7** [18] were the only products obtained (cf. Scheme 2).

The methylene group in *p*-nitrobenzylcyanide (**8a**) was sufficiently acidic to couple directly with benzenediazonium chlorides to yield **1a**–**c** and thus initial conversion into enamine was not needed. Condensation of **8a** with dimethylformamide dimethylaceteal (DMFDMA) under reflux for 3 h. produced **9a** in 70% yield.

**Scheme 3:**
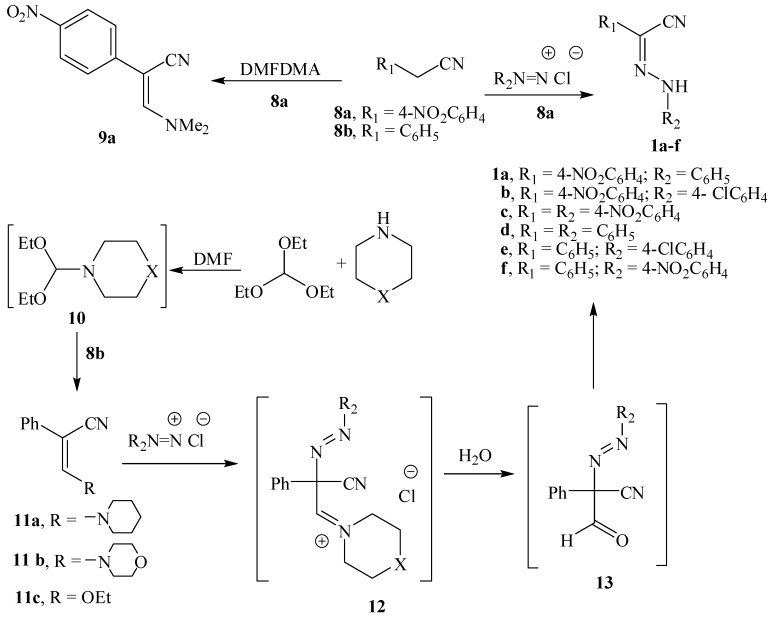
Preparation of hydrazones **1a**–**f**.

Trials to condense benzyl cyanide (**8b**) with DMFDMA to obtain **9b** failed, as the volatility of the reagent did not allow for the use of high reaction temperatures. Consequently we decided to generate less volatile formamide acetals *in situ* thus allowing for employing higher temperatures. We could thus successfully prepare **11a**,**b** via refluxing triethyl orthoformate, piperidine or morpholine with benzyl cyanide in dimethylformamide solution under reflux for 72 h. (cf. Scheme 3) [22]. Although it is possible that the initially formed ethoxyacrylonitrile (**11c**) reacted with the secondary amine, this possibility could be readily excluded as benzyl cyanide failed to condense with triethyl orthoformate under reflux with DMF in the absence of secondary amine. We thus believe that the acetals **10a**,**b** are initially formed and then these react with benzyl cyanide to yield **11a**,**b**. This condensation could be effected by microwave heating for 20 min to obtain **11a**,**b**, and were found identical in all details (melting point and TLC analysis, NMR) to the compounds obtained under conventional heating.

We have decided to test the possibility that enaminonitriles **11** can couple with aromatic diazonium salts to yield intermediates **12** that would be readily converted into **13** and consequently into **1d**–**f** [23] via a Japp-Klingmann cleavage [24], a similar coupling process that has been previously reported by us [15].

Enaminonitriles **1** were found to be good candidates to obtain 4-aminopyrazoles based on a Thorpe-Ziegler cyclization [25,26]. In this method, * N*-alkylation of enaminonitrile was carried out using *α*-haloketones in anhydrous DMF in the presence of K_2_CO_3_ as the base. Moreover, compounds having an aryl substituent on the amino moiety of the enamine group were the most convenient for alkylation and spontaneous intramolecular cyclization. The presence of this group facilitates the formation of the *N*-anion required for alkylation and subsequent carbanion formation for the cyclization involving the cyano group. The reaction of enaminonitrile **1** with chloroacetonitrile, chloroacetone, ethyl bromoacetate and *α*-bromoacetophenone in DMF/K_2_CO_3_ afforded the corresponding 4-aminopyrazole derivatives **3a**–**e** in low yield (27–44%) *via *intermediate **2**. We prepared compound **3** by a modification of the method used by Gewald *et al*. [24,25] using triethylamine as base. When the reaction was carried out in an excess of triethylamine solution, the desired 4-aminopyrazole derivatives **3a**–**e** are obtained in a satisfactory yield (77–92%).

The structure of compounds **3a**–**e**was established on the basis of elemental analysis, IR, mass, ^1^H and ^13^C-NMR spectral data studies (*cf*. Experimental Section). For example, the ^1^H-NMR spectrum of compound **3a** showed the absence of a signal for a methylene function and the presence of a two proton D_2_O-exchangeable signal at *δ *= 6.45 ppm for the amino function and the aromatic protons in the proper positions. ^13^C-NMR and mass spectra of compound **3a** are in agreement with the proposed structure. This synthesis thus opens a new route for synthesis of 4-aminopyrazole-5-carbonitriles; important intermediates in synthesis of pharmaceuticals (e.g., Viagra). The scope of this synthetic approach is being now explored.

## 3. Experimental

### 3.1. General

All melting points are uncorrected. IR spectra were recorded in KBr with a Bruker Vector 22 (Ettlingen, Germany) spectrophotometer. The ^1^H-NMR (300 MHz) and ^13^C-NMR (75.4 MHz) spectra were recorded on a Varian Mercury 300 MHz spectrometer in DMSO-d_6_ as solvent and TMS as internal standard; chemical shifts are reported in δ units (ppm). Mass spectra were measured at 70 eV using a Shimadzu GCMS-QP-1000 EX mass spectrometer. Microanalyses were performed on a LECO CHN-932 by the Microanalysis Unit of Cairo University. Microwave experiments were conducted in aCEM MARS oven.

### 3.2. Preparation of Substituted Aryl Hydrazonoacetonitriles **1a–f**

A cold solution of aryldiazonium salt (10 mmol) was prepared by adding a solution of sodium nitrite (10 mmol in H_2_O) to a cold solution of the aromatic amine hydrochloride with stirring. The resulting solution of the aryldiazonium salt was added to a cold solution of *p*-nitrobenzylcyanide (**8a**, 1.62 g, 10 mmol), 2-phenyl-3-piperidin-1-yl-acrylonitrile (**11a**) or 3-morpholin-4-yl-2-phenyl-acrylonitrile (**11b**) in ethanol (50 mL) containing sodium acetate (5 g). The reaction mixture was stirred at room temperature for 30 min. The solid product, so formed, was collected by filtration, washed with water and crystallized from the appropriate solvent.

*4-Nitrophenyl(phenylhydrazono)acetonitrile* **(1a**). Yield 70%. m.p. 200–201 °C (lit. m.p. = 198 °C [23]). IR: *ν* = 3380 (NH), 2208 (CN), 1463, 1376 (NO_2_) cm^−1^. ^1^H-NMR: *δ* = 6.99–7.25 (m, 5-H, Ar-H), 7.30 (s, 1H, NH), 7.75 (d, 2H, *J* = 10 Hz, Ar-H), 8.22 (d, 2H, *J* = 10 Hz, Ar-H). ^13^C-NMR: *δ* = 157.2 (C=N), 155.2, 144.1, 137.4, 130.2, 129.1, 124.5, 123.4, 120.2, 117.2 (CN). MS (EI): *m/z* (%) = 266 (M^+^, 89%). Anal. Calcd. for C_14_H_10_N_4_O_2_ (266.25): C, 63.15; H, 3.79; N, 21.04. Found: 63.05; H, 3.80; N, 21.10.

*(4-Chlorophenyl)hydrazono(4-nitrophenyl)acetonitrile* (**1b**). Yield 79%. m.p. 225–227 °C. IR: *ν* = 3395 (NH), 2218 (CN), 1460, 1372 (NO_2_) cm^−1^. ^1^H-NMR: *δ* = 6.80 (d, 2H, *J* = 9.5 Hz, Ar-H), 7.20 (d, 2H, *J* = 9.5 Hz, Ar-H), 7.35 (s, 1H, NH), 7.56 (d, 2H, *J* = 10 Hz, Ar-H), 8.29 (d, 2H, *J* = 10 Hz, Ar-H). MS (EI): *m/z* (%) = 300 (M^+^, 65%). Anal. Calcd. for C_14_H_9_ClN_4_O_2_ (300.70) C, 55.92; H, 3.02; Cl, 11.79; N, 18.63. Found: 55.80; H, 3.00; Cl, 11.90; N, 18.55.

*(4-Nitrophenyl)-(4-nitrophenyl)hydrazonoacetonitrile* (**1c**). Yield 75%. m.p. 235–236 °C. IR: *ν* = 3385 (NH), 2220 (CN); 1466, 1379 (NO_2_) cm^−1^. ^1^H-NMR: *δ* = 6.90 (d, 2H, *J* = 10 Hz, Ar-H), 7.10 (s, 1H, NH), 7.65 (d, 2H, *J* = 10 Hz, Ar-H), 7.86 (d, 2H, *J* = 10 Hz, Ar-H), 8.15 (d, 2H, *J* = 10 Hz, Ar-H). MS (EI): *m/z* (%) = 311 (M^+^, 87%). Anal. Calcd. for; C_14_H_9_N_5_O_4_ (311.25): C, 54.02; H, 2.91; N, 22.50. Found: C, 53.90; H, 2.80; N, 22.45.

*Phenyl(phenylhydrazono)acetonitrile* (**1d**). Yield 68%, m.p. 100–102 °C. IR: *ν* = 3360 (NH), 2210 (CN) cm^−1^. ^1^H-NMR: *δ* = 6.99–7.25 (m, 5-H, Ar-H), 7.30 (s, 1H, NH), 7.40–7.62 (m, 5-H, Ar-H). MS (EI): *m/z* (%) = 221 (M^+^, 89%). Anal. Calcd. for C_14_H_11_N_3_ (221.26): C, 76.00; H, 5.01; N, 18.99. Found: 75.90; H, 5.10; N, 19.05.

*(4-Chlorophenyl)hydrazonophenyl acetonitrile* (**1e**). Yield 82%, m.p. 180–182 °C (lit. m.p. = 168 °C [23]). IR: *ν* = 3360 (NH), 2215 (CN) cm^−1^. ^1^H-NMR: *δ* = 7.00 (d, 2H, *J* = 10 Hz, Ar-H), 7.18 (d, 2H, *J* = 10 Hz, Ar-H), 7.28 (s, 1H, NH), 7.35–7.65 (m, 5-H, Ar-H). MS (EI): *m/z* (%) = 255 (M^+^, 81%). Anal. Calcd. for C_14_H_10_ClN_3_ (255.70): C, 65.76; H, 3.94; Cl, 13.86; N, 16.43. Found: C, 65.70; H, 3.80; Cl, 13.90; N, 16.55.

*(4-Nitrophenyl)hydrazonophenylacetonitrile* (**1f**). Yield 77%, m.p. 212–214 °C (lit. m.p. = 214 °C [23]). IR: *ν* = 3385 (NH), 2220 (CN) 1460, 1372 (NO_2_) cm^−1^; ^1^H-NMR: *δ* = 6.95 (d, 2H, *J* = 10 Hz, Ar-H), 7.07 (s, 1H, NH), 7.25–7.68 (m, 5H, Ar-H), 7.92 (d, 2H, *J* = 10 Hz, Ar-H). MS (EI): *m/z* (%) = 266 (M^+^, 91%). Anal. Calcd. for C_14_H_10_N_4_O_2_ (266.25): C, 63.15; H, 3.79; N, 21.04. Found: C, 63.20; H, 3.85; N, 21.15.

### 3.3. General Procedure for Preparation of 4-Aminopyrazole Derivatives **3a–e**

*Method A*. A mixture of **1a**,**f** (0.01 mol), the α-halo compound (chloroacetonitrile, chloroacetone, ethyl bromoacetate and α-bromoacetophenone, 0.011 mol), and potassium carbonate (2.0 g) in dimethylformamide (20 mL) was stirred for 1 h, at 90 °C, in an oil-bath. The reaction mixture was cooled and poured into water (60 mL). The precipitated solid products formed were filtered off, washed thoroughly with cold water and recrystallized from EtOH to afford the corresponding cyclized products **3a** (40 %), **3b** (47%), **3c** (39%), **3d** (35%), **3e** (22%).

*Method B*. To a solution of the intermediate **1a**,**f** (0.01 mol) the α-halo compound (chloroacetonitrile, chloroacetone, ethyl bromoacetate and α-bromoacetophenone, 0.011 mol) and triethylamine (4 mL) were added with external cooling. The reaction mixture was refluxed for 20–30 min, after cooling (50 mL) water was added, the solid product was filtered off, washed thoroughly with cold water and crystallized from ethanol (in the case of **3a**, 88%, **3b**, 92). For derivatives **3c**–**e** a brown oil was separated, the water was decanted and the oil was extracted with CH_2_Cl_2_ (3 × 25 mL) and the combined organic layers were dried (Na_2_SO_4_), filtered and the solvent was evaporated to give a solid which was crystallized from EtOH.

*4-Amino-3-(4-nitrophenyl)-1-phenyl-1H-pyrazole-5-carbonitrile* (**3a**). Yield 88%, m.p. 187–188 °C; IR: *ν* = 3310–3250 (NH_2_), 2224 (CN), 1463, 1375 (NO_2_) cm^−1^; ^1^H-NMR: *δ* = 6.45 (s, 2H, NH_2_), 7.25–7.38 (m, 5H, Ar-H), 7.62 (d, 2H, *J* = 10 Hz, Ar-H), 8.10 (d, 2H, *J* = 10 Hz, Ar-H); ^13^C-NMR: *δ* = 98.98 (C-5), 113.23 (CN), 116.13 (C-2′, 6′), 123.38 (C-3′′, 5′′), 123.71 (C-4′), 129.38 (C-2′′, 6′′), 129.82 (C-3′, 5′), 140.05 (C-1′), 144.77 (C-1′′), 145.23 (C-3), 146.90 (C-4), 147.65 (C-4′′). MS (EI): *m/z* (%) = 305 (M^+^, 100%). Anal. Calcd. for C_16_H_11_N_5_O_2_ (305.): C, 62.95; H, 3.63; N, 22.94. Found: C, 63.10; H, 3.85; N, 23.05.

*4-Amino-1-(4-nitrophenyl)-3-phenyl-1H-pyrazole-5-carbonitrile* (**3b**). Yield 92%, m.p. 178–180 °C IR: *ν* = 3320–3240 (NH_2_), 2228 (CN) 1466, 1369 (NO_2_) cm^−1^, ^1^H-NMR: *δ* = 6.49 (s, 2H, NH_2_), 7.25–7.48 (m, 5H, Ar-H), 7.55 (d, 2H, *J* = 10 Hz, Ar-H), 8.20 (d, 2H, *J* = 10 Hz, Ar-H). MS (EI): *m/z* (%) = 305 (M^+^, 85%). Anal. Calcd. for C_16_H_11_N_5_O_2_ (305): C, 62.95; H, 3.63; N, 22.94. Found: C, 63.10; H, 3.85; N, 23.05. Found: C, 63.00; H, 3.65; N, 22.80.

*Ethyl 4-amino-1-(4-nitrophenyl)-3-phenyl-1H-pyrazole-5-carboxylate* (**3c**). Yield (74%), m.p. 165–166 °C; IR: ν = 3492–3380 (NH_2_), 1715 (C=O) cm^−1^. ^1^H-NMR: δ = 0.98 (t, 3H, *J* = 7.5Hz, CH_3_), 4.04 (q, 2H, *J* = 7.5 Hz, CH_2_), 5.94 (s, 2H, NH_2_), 7.21 (d, 2H, *J* = 9 Hz, Ar-H), 7.28 (t, 1H, *J* = 8 Hz, Ar-H), 7.31–7.39 (m, 4H, Ar-H), 8.14 (d, 2H, *J* = 9 Hz, Ar-H). ^13^C-NMR: δ = 14.12 (CH_3_), 59.10 (CH_2_), 108.72 (C-5), 113.53 (C-3′,5′), 121.23 (C-2′′,6′′), 127.21 (C-2′,6′), 127.94 (C-4′′), 132.14 (C-1′), 132.92 (C-3′′,5′′), 137.31 (C-1′′), 141.96 (C-3), 146.41 (C-4), 150.86 (C-4′), 160.32 (C=O). MS (EI): *m/z* (%) = 352 (M^+^, 77%). Anal. Calcd. For C_18_H_16_N_4_O_4_ (352.12): C, 61.36; H, 4.58; N, 15.90. Found C, 61.55; H, 4.42; N, 16.12.

*1-(4-Amino-1-(4-nitrophenyl)-3-phenyl-1H-pyrazol-5-yl)ethanone* (**3d**). Yield: 77%, m.p. 192–193 °C. IR: ν = 3440–3348 (NH_2_), 1678 (C=O) cm^−1^. ^1^H-NMR: *δ* = 2.23 (s, 3H, CH_3_), 6.67 (s, 2H, NH_2_), 7.09 (t, 1H, *J* = 9 Hz, Ar-H), 7.36 (d, 2H, *J* = 9 Hz, Ar-H), 7.42–7.55 (m, 4H, Ar-H), 8.23 (d, 2H, *J* = 9 Hz, Ar-H). ^13^C-NMR: *δ* = 28.52 (*C*H_3_), 114.28 (C-3′,5′), 118.20 (C-5), 122.12 (C-2′′,6′′), 127.93 (C-2′,6′), 128.51 (C-4′′), 132.21 (C-1′), 133.62 (C-3′′,5′′), 138.64 (C-1′′), 143.31 (C-3), 147.45 (C-4), 149.38 (C-4′), 186.21 (C=O). MS (EI): *m/z *(%) = 332 (80) [M]^+^. Anal. Calcd. For C_17_H_14_N_4_O_3_ (322.32): C, 63.35; H, 4.38; N, 17.38. Found C, 63.49; H, 4.17; N, 17.15.

*(4-Amino-1-(4-nitrophenyl)-3-phenyl-1H-pyrazol-5-yl)(phenyl)methanone* (**3e**). Yield: 72%; m.p. 224–226 °C. IR: ν = 3452–3367 (NH_2_), 1690 (C=O) 1465, 1372 (NO_2_) cm^−1^. ^1^H-NMR: *δ* = 6.58 (s, 2H, NH_2_), 7.14 (d, 2H, *J* = 9 Hz, Ar-H), 7.20–7.26 (m, 4H, Ar-H), 7.30–7.38 (m, 6H, Ar-H), 8.12 (d, 2H, *J* = 9 Hz, Ar-H). MS (EI): *m/z *(%) = 384 (42) [M]^+^. Anal. Calcd. For C_22_H_16_N_4_O_3_ (384.39): C, 68.74; H, 4.20; N, 14.58. Found C, 68.60; H, 4.44; N, 14.70.

### 3.4. 3-Dimethylamino-2-(4-nitrophenyl) acrylonitrile (**9a**)

A mixture of **8a** (0.01 mol) and DMFDMA (0.012 mol) in dioxane (15 mL) was refluxed for three hours then cooled and poured onto water. The green solid product, so formed, was collected by filtration and crystallized from ethanol to give **9a**. Yield 70%; m.p. 178–180 °C. IR: *ν* = 2222 (CN), 1610 (C=C) cm^−1^. ^1^H-NMR: *δ* = 3.27 (s, 3H, CH_3_), 3.33 (s, 3H, CH_3_), 7.50 (d, 2H, *J* = 9Hz, Ar-H), 7.80 (s, 1H, olefinic-H), 8.12 (d, 2H, *J* = 9Hz, Ar-H). ^13^C-NMR: *δ* = 43.64 (2*C*H_3_), 97.27 (*C*=CH), 122.54 (CN), 123.36, 132.40, 144.45, 145.75, 150.50 (C=*C*H). MS (EI): *m/z* (%) = 217 (100) [M^+^]. Anal. Calcd. For C_11_H_11_N_3_O_2_ (217.22): C, 60.82, H, 5.10, N, 19.34. Found C, 60.85, H, 5.03, N, 19.30.

### 3.5. 3-Substituted Amino-2-phenylacrylonitriles **11a,b**

*Method A*. To a mixture of benzyl cyanide **8b** (0.3 mol), triethyl orthoformate (0.32 mol), and piperidine or morpholine (0.3 mol each) DMF (40 ml) was added and the solution was refluxed for 72 h. The reaction mixture was then cooled and poured onto water. The solid product formed, was collected by filtration and crystallized from ethanol, (**11a** 70%; **11b** 65%).

*Method B*. *Under Microwave irradiation. *In a round bottom flask of 100 mL equipped with a condenser, benzyl cyanide **8b** (0.3 mol), triethyl orthoformate (0.32 mol), and piperidine or morpholine (0.3 mol each) DMF (40 ml) was added and the mixture was heated at reflux during 20 min under microwave irradiation (at a constant power of 400 W). After cooling to r.t., the reaction mixture was poured onto water to give a solid, which was identical in all respects with that obtained from the above reactions (TLC, m.p., NMR), (**11a** 85%; **11b** 77%).

*2-Phenyl-3-piperidin-1-yl-acrylonitrile * (**11a**). Yield: 85%, m. p. 116–117 °C. IR: *ν* = 2190 (CN), 1616 (C=C) cm^−1^. ^1^H-NMR: *δ* = 1.60 (s, 6H, 3CH_2_), 3.63 (s, 4H, 2CH_2_), 7.16 (s, 1H, olefinic-H), 7.26–7.45 (m, 5 H, Ar-H). ^13^C-NMR: *δ* = 24.36, 26.41, 51.96, 75.41 (*C*=CH), 121.59 (CN), 124.48, 125.51, 129.14, 137.27, 149.29 (C=*C*H). MS (EI): *m/z* (%) = 212 (42) [M^+^]. Anal. Calcd. For C_14_H_16_N_2_ (212.29): C, 79.21, H, 7.60, N, 13.20. Found C, 79.29, H, 7.67, N, 13.17.

*3-Morpholin-4-yl-2-phenyl-acrylonitrile* (**11b**). Yield 77%, m.p. 105–106 °C; IR: *ν* = 2205 (CN), 1620 (C=C). ^1^H-NMR: *δ* = 2.85 (t, 4H, 2CH_2_), 3.75 (t, 4H, 2CH_2_), 6.92 (s, 1H, olefinic-H), 7.15–7.39 (m, 5H, Ar-H). MS (EI): *m/z* 214 (M^+^, 93%). Anal. Calcd. For C_13_H_14_N_2_O_2_ (214.): C, 72.87; H, 6.59; N, 13.07. Found: C, 72.80; H, 6.55; N, 13.10.

## 4. Conclusions

Enaminonitriles could be easily obtained by reaction of benzyl cyanide or 4-nitrobenzyl cyanide with DMFDMA or with triethylorthoformate and piperidine or morpholine in the presence of DMF, *in situ* generation of less volatile amide acetals from piperidine diethylacetal would enable avoiding the application of drastic reaction conditions in the condensation of amide acetals with active methylenes, thus further application of condensation reaction to less reactive methylenes can be applied. This method involves the of less expensive chemicals and conversion of methylenes to enamines enhances reactivity toward electrophiles. Shorter reaction times and higher yields were obtained by microwave irradiation.
